# Clinical value of Melanoma Inhibitory Activity in stratifying malignant melanoma patients

**DOI:** 10.1186/1479-5876-13-S1-P10

**Published:** 2015-01-15

**Authors:** Angela Sandru, Silviu Voinea, Eugenia Panaitescu, Madalina Bolovan, Adina Stanciu, Sabin Cinca, Alexandru Blidaru

**Affiliations:** 1 Department of Surgical Oncology, “Carol Davila” University of Medicine and Pharmacy; “Alexandru Trestioreanu” Oncologic Institute, Bucharest, Romania; 2 Department of Medical Informatics and Biostatistics ,“Carol Davila” University of Medicine and Pharmacy, Bucharest, Romania; 3 Department of Carcinogenesis and Molecular Biology, “Alexandru Trestioreanu” Oncologic Institute, Bucharest, Romania

## Background

Malignant melanoma (MM) is a heterogeneous disease, well-known for its unpredictable clinical course and lack of response to most systemic therapies. Furthermore in some parts of the world it has quite an epidemic nature, with an incidence that has increased steadily in the last 30 years [1]. Therefore the characterization of new tumor markers that could better suggest the patient outcome has become a priority for many research centers. Melanoma Inhibitory Activity (MIA) is a protein highly expressed and secreted by malignant melanocytes [1,2], without being identified in benign melanocytes or other normal skin cells [3]. More, the induction of MIA synthesis seems to be an early event in carcinogenesis, taking into account that all in situ tumors express it. The objective of this study was to assess the significance of increased serum MIA concentration in MM patients with no metastases at primary diagnoses.

## Materials and methods

Between 2008-2012 we have collected 200 blood samples from 150 patients with non-metastatic MM and 50 healthy donors. The patients were staged according to TNM classification 2009 as follows: 28 in stage I, 72 in stage II and 50 in stage III. The blood was withdrawn after the primary tumor excision and before any other treatment. MIA was measured by a high sensitivity ELISA method, with a kit produced by Roche Diagnostics. Using the ROC curve, MIA cut-off value was set at 9.4 ng/ml [4].

Results We estimated overall survival (OS) and disease free survival (DFS) for the entire lot and for each stage separately, according to MIA cut-off level. The length of follow-up was 44 month, from the moment of MIA measurement. Univariate Cox regression suggested that patients with an increased MIA serum concentration had a three times higher risk of relapse (HR=3.3895) and death (HR = 2.7597) than patients with values under the calculated threshold (p=0.000). More important is that MIA keeps statistical significance in multivariate analyses, predicting risk of death or relapse after corrections for clinical stage.

## Conclusions

In our study, MM patients with a MIA serum concentration above 9.4 ng/ml had a worse DFS (p=0.0109) and OS (p=0.0009) than patients with values below 9.4 ng/ml. We consider that MIA serum concentration is a valuable prognostic factor and could become a tool for selecting patients at risk for developing metastases rendering them eligible for neoadjuvant treatment.
